# Two-step pipeline for oral diseases detection and classification: a deep learning approach

**DOI:** 10.3389/froh.2025.1659323

**Published:** 2025-10-27

**Authors:** Anna Luíza Damaceno Araújo, Arnaldo Vitor Barros da Silva, Ana Rita Marega Gonçalves, Cristina Saldivia-Siracusa, Daniel Lobato Ferreira Ferraz, Camila Barcellos Calderipe, Ivan José Correia-Neto, Pablo Agustin Vargas, Marcio Ajudarte Lopes, Paulo Rogério Ferreti Bonan, André Carlos Ponce de Leon Ferreira de Carvalho, Marcos G. Quiles, Alan Roger Santos-Silva, Luiz Paulo Kowalski

**Affiliations:** 1 Head and Neck Surgery Department and LIM 28, University of São Paulo Medical School, São Paulo, São Paulo, Brazil; 2 Institute of Science and Technology, Federal University of São Paulo, São José dos Campos, São Paulo, Brazil; 3 Institute of Mathematics and Computer Sciences, University of São Paulo, São Carlos, São Paulo, Brazil; 4 Faculdade de Odontologia de Piracicaba, Universidade Estadual de Campinas (FOP-UNICAMP), Piracicaba, São Paulo, Brazil; 5 Department of Clinical and Social Dentistry, Health Sciences Center, Federal University of Paraíba-UFPB, João Pessoa, Paraíba, Brazil; 6 Department of Head and Neck Surgery and Otorhinolaryngology, A.C. Camargo Cancer Center, São Paulo, São Paulo, Brazil

**Keywords:** object detection, image classification, artificial intelligence, pre-training, oral potentially malignant disorders, oral squamous cell carcinoma

## Abstract

**Introduction:**

This study aimed to develop and evaluate an artificial intelligence pipeline combining object detection and classification models to assist in early identification and differentiation of oral diseases.

**Methods:**

This retrospective cross-sectional study utilized clinical images of oral potentially malignant disorders and oral squamous cell carcinoma, comprising a baseline dataset of 773 images from Faculdade de Odontologia de Piracicaba, Universidade Estadual de Campinas (FOP-UNICAMP) and an external validation dataset of 132 images from Federal University of Paraíba (UFPB). All images were obtained prior to biopsy, all with corresponding histopathological reports. For object detection, ten YOLOv11 models were developed with varying data augmentation strategies, trained for 200 epochs using pretrained COCO weights. For classification, three MobileNetV2 models were trained on images cropped according to the experts' reference bounding box annotations, each using different combinations of learning rates and data augmentation. After selecting the best detector–classifier combination, we integrated them into a two-step pipeline in which the images cropped by the detector were subsequently forwarded to the classifier.

**Results:**

The best YOLOv11 configuration achieved a mAP50 of 0.820, precision of 0.897, recall of 0.744, and F1-score of 0.813. For classification, the best MobileNetV2 configuration achieved an accuracy of 0.846, precision of 0.871 recall of 0.846, F1-score of 0.844, and AUC-ROC of 0.852. On external validation, this same model reached an accuracy of 0.850, precision of 0.866, recall of 0.850, F1-score of 0.851, and an AUC-ROC of 0.935. The two-step approach, when applied to the test set from the baseline dataset, achieved an accuracy of 0.784, precision of 0.793, recall of 0.784, F1-score of 0.784, and an AUC-ROC of 0.811. When evaluated on the external validation dataset, it yielded an accuracy of 0.863, precision of 0.879, recall of 0.863, F1-score of 0.866, and an AUC-ROC of 0.934. The visual inspection of YOLO's inference outputs confirmed consistent lesion localization across diverse oral cavity images, with some missing (17.4%). The t-SNE visualization demonstrated partial separation between oral potentially malignant disorder (OPMD) and oral squamous cell carcinoma (OSCC) feature embeddings, indicating the model captured discriminative patterns with some class overlap.

**Conclusion:**

This proof-of-concept study demonstrates the feasibility of a two-step artificial intelligence (AI) pipeline combining object detection and classification to support early diagnosis of oral diseases. However, caution is warranted when interpreting the results of two-step approaches, as images missed by YOLO during detection are excluded from the classification stage, which may affect the reported performance metrics.

## 1 Introduction

Early detection of oral cancerous and precursor lesions is essential to reduce diagnoses at advanced stages, significantly improving treatment outcomes and prognosis and delaying potential malignant transformation (1). However, delays in diagnosis are common, mainly because, in the initial stages, oral potentially malignant disorders (OPMDs) often present as asymptomatic and smooth-surfaced lesions that do not raise suspicion among patients. Such initial lesions may be clinically indistinguishable from indolent forms of oral squamous cell carcinoma (OSCC), the socalled incipient lesions, which present as plaques in up to 80% of cases (2). The lack of awareness about early signs contributes to late diagnosis by general practitioners and delays in seeking care by patients. Additionally, barriers to accessing specialists in remote regions further exacerbate the problem, limiting opportunities for timely assessment and intervention.

Currently, in the context of oral cancer, conventional oral examination is the only widely used resource for early identification of lesions (3), which depends heavily on the examiner's experience and vigilance. According to Tarakji et al. (4) the diagnosis of OPMDs depends on adequate clinical skills and histological investigation with specialists showing better knowledge than general practitioners. To help fill this gap, investing in continuing education and training, as well as developing support systems to aid clinicians in identifying early lesions, are promising strategies. In this regard, many alternative testing and diagnostic aids have been proposed to improve early detection and risk assessment. Among them, artificial intelligence (AI) approaches based on clinical photographs have the potential to simplify the diagnostic workflow by applying computer vision techniques to distinguish OPMD from malignant lesions (5, 6), accelerate expert referral (7) and biopsy procedures, and help overcome the limitations of conventional oral examination, which relies mainly on clinical indicators of malignancy, such as increased redness and ulceration (8, 9) that are sometimes present in OPMDs, creating diagnostic uncertainty. Therefore, when training convolutional neural networks (CNNs) with white-light photographs, two main tasks are usually explored: object detection for lesion localization and image classification. (10) The exploration of both detection and classification strategies is fundamental for the development of robust AI-based diagnostic frameworks.

Object detection refers to a process in computer vision that determines where objects are located in a given image, typically by drawing bounding boxes around them, and identifying which class each object belongs to (11). Such algorithms stand out as innovative tools to support clinical decision-making, as they allow for precise localization of suspicious regions within oral photographs. This minimizes the risk of missing subtle abnormalities and ensures that clinically relevant areas are systematically analyzed, while also enabling their incorporation into pipelines for lesion identification and subsequent classification. The main architectures explored in this oral disease's context include YOLO, Faster R-CNN, RetinaNet, and CenterNet2. These models are particularly valuable for identifying smooth-surfaced leukoplakias, which could otherwise go unnoticed during routine examinations. Nonetheless, important limitations have been reported in the literature, such as reliance on limited datasets, absence of external validation, and challenges in detecting small lesions (12–16).

Classification algorithms, in turn, assign diagnostic labels to entire images or regions of interest without necessarily determining lesion boundaries. By learning patterns in clinical photographs, these models can distinguish between benign, potentially malignant, and malignant conditions, thereby guiding referrals and supporting clinical decision-making, particularly in resource-limited settings. In oral diseases, CNN-based classification has been increasingly explored to automate diagnosis and prioritize cases for specialist evaluation (5, 17–23). Despite their potential, classification algorithms also face challenges related to dataset representativeness, interpretability, and integration into clinical workflows.

The aim of this research is to develop detection and classification algorithms to assist in the early detection of OPMD and OSCC and further integrate them in a two-step pipeline enhancing the reliability of classification by restricting the analysis to pertinent regions, whereas classification provides clinical interpretation to the detected areas. This sequential approach not only strengthens diagnostic performance but also reflects the conventional workflow of clinicians, who initially identify lesions before assessing their malignant potential, thereby increasing both the accuracy and interpretability of the proposed model.

## 2 Materials and methods

### 2.1 Dataset

This retrospective cross-sectional study was developed based on a real-world dataset comprising 773 clinical photographs collected from patients diagnosed with oral lesions at Faculdade de Odontologia de Piracicaba, Universidade Estadual de Campinas (Piracicaba, São Paulo, Brazil) between 2000 and 2025. Images were categorized as OPMD (*n* = 380) and OSCC (*n* = 393). For external validation, an independent subset comprising 53 OPMD and 79 OSCC images from patients at the Federal University of Paraíba (UFPB) (João Pessoa, Paraíba, Brazil) was included. Both categories were defined according to the clinical and histopathological criteria established by the World Health Organization (WHO Classification of Tumours Editorial Board, 2022). The OPMD category included proliferative verrucous leukoplakia and conventional leukoplakia (with or without oral epithelial dysplasia), while the OSCC category comprised several clinical and histopathological variants (i.e., conventional, verrucous, and incipient) to increase dataset variability. To ensure consistency in imaging and diagnostic labels, we excluded images of poor quality or those from non-representative biopsies, defined as (1) biopsies with a histopathological diagnosis of OED despite clear clinical features of OSCC, or (2) biopsies that were too small or technically inadequate to permit a definitive diagnosis. In cases with significant clinical changes prompting a repeat biopsy, only images acquired prior to each biopsy were included, provided there was a minimum interval of three months between procedures. All images were obtained before biopsy and had corresponding histopathological reports. The dataset was non-randomly divided into independent subsets of training, validation and testing, in which photos from the same patient were kept in the training subset to avoid data leakage, and proportions of the main class were followed in the subsets (Table 1).

**Table 1:** Datasets.

Classes	Baseline dataset (FOP-UNICAMP)				External validation dataset (UFPB)
	Training (80%)	Validation (10%)	Test (10%)	Total	
OPMD	312	34	34	380	53
OSCC	316	39	38	393	79
Total	328	73	72	773	132

OPMD, oral potentially malignant disorder; OSCC, oral squamous cell carcinoma.

Bounding box annotations were made by A.L.D.A., in consultation with C.S.S. to reach a consensus, using Aperio ImageScope software (Leica Biosystems) and a Huion Inspiroy H1060P graphics tablet, blinded to the diagnosis, and focusing on framing the lesions within rectangular boxes.

This study was conducted in accordance with the Checklist for Artificial Intelligence in Medical Imaging (CLAIM) (24) and the Must AI Criteria-10 (MAIC-10) Checklist (25) (Supplementary Table S1). It adhered to the principles of the Declaration of Helsinki and received ethical approval from the Piracicaba Dental School Ethical Committee (Registration number: 42235421.9.0000.5418) and from the Federal University of Paraíba Ethical Committee (Registration number: 72314323.0.0000.5188). The approvals also included Material Transfer Agreements between participating institutions to facilitate the sharing of images.

### 2.2 Workstation

All experiments were conducted on Google Colab using a standardized virtualized environment with an Intel(R) Xeon(R) CPU @ 2.00 GHz (2 threads, 1 physical core), 39 MB of L3 cache. The system was also equipped with an NVIDIA Tesla T4 GPU (15,360 MiB VRAM, CUDA 12.4, Driver 550.54.15).

### 2.3 Object detection task

YOLO (You Only Look Once) (26) is one of the most efficient algorithms for object detection in images, based on convolutional neural networks (CNNs). Unlike traditional approaches that analyze the image in several steps or propose regions of interest before classification, YOLO treats detection as a single regression problem, where the entire image is processed at once. The model divides the image into a grid and, for each cell of the grid, it provides bounding boxes, class probabilities and associated confidence gains. This structure allows YOLO to be extremely fast, enabling real-time applications, without significantly compromising accuracy. In addition, its end-to-end training capability (end-to-end learning) makes the process more efficient and straightforward, being widely used in tasks such as security monitoring, medical imaging diagnosis and driving.

YOLOv11 (27), the latest iteration in the YOLO series, introduces several architectural innovations aimed at enhancing performance across various computer vision tasks. Key advancements include the incorporation of the C3k2 (Cross Stage Partial with kernel size 2) block, SPPF (Spatial Pyramid Pooling—Fast), and C2PSA (Convolutional block with Parallel Spatial Attention) components. These additions contribute to improved feature extraction and overall model efficiency.

Ten models were developed using four different YOLOv11 architecture variants: YOLOv11n (2.6M parameters), YOLOv11s (9.4M), YOLOv11m (20.1M), and YOLOv11l (25.3M). Each configuration was combined with specific data augmentation strategies to evaluate their impact on performance. All versions were initialized with COCO's (28) pretrained weights and trained for 200 epochs using images resized to 640 × 640 pixels. The following augmentations were applied: hue adjustment (±0.015), saturation adjustment (±0.7), image translation (±10% of the image size), and scaling (±50%). Horizontal flipping was also applied with a probability of 0.5. Additional variations were included, such as mosaic augmentation, which randomly combines one to four images during training.

In object detection experiments, the mean Average Precision at 50% Intersection over Union (mAP50) was used as the primary evaluation metric, emphasizing accurate localization of lesion regions. A single target class, “lesion” (merging OPMD and OSCC), was adopted because performance would not be adversely affected, and preliminary results showed that while the model was effective at locating lesions, it was significantly less capable of distinguishing between their specific types. When both classes were evaluated separately, the mAP50 dropped to approximately 22%. Consequently, calculation of the AUROC was considered inappropriate, as the task effectively constitutes a single-class problem. Additionally, Precision, Recall, and F1-Score were computed using the built-in evaluation tools provided by the Ultralytics library.

Although mAP50 was chosen as our primary metric for consistency with common object detection literature, it is important to note that for a single-class problem, mAP50 is equivalent to the Average Precision (AP) at IoU = 0.5. Furthermore, AP is formally defined as the area under the precision-recall curve (AUC-PR). Thus, AP and AUC-PR are synonymous metrics; reporting both would be redundant. We have chosen to report AP to align with the standard reporting conventions in our field.

### 2.4 Classification task

Three models based on MobileNetV2 (29, 30) architecture were developed with distinct learning rates and data augmentation strategies. The classifiers were trained on images cropped according to the experts' reference bounding box annotations, not the whole image. The learning rate values were selected through systematic hyperparameter tuning, guided by the observed decay in loss during preliminary experiments. All versions were initialized with ImageNet (31, 32) pretrained weights and trained for 200 epochs with image sizes of 224 × 224 pixels. The following augmentations were applied: brightness, contrast, and saturation adjustments (±0.2), hue adjustment (±0.1), and image translation (±10% of the image size). Random horizontal and vertical flipping were implemented with some variations. Since the classes were perfectly equal, no strategies to account for class imbalance were conducted.

Performance metrics as accuracy, precision, recall, F1-score and the Area Under the Receiver Operating Characteristic Curve (AUC-ROC) were computed using the scikit-learn library (33). After selecting the best detector–classifier combination, we integrated them into a two-step pipeline in which the images cropped by the detector were subsequently forwarded to the classifier.

## 3 Results

### 3.1 Object detection

**Figure 1:**
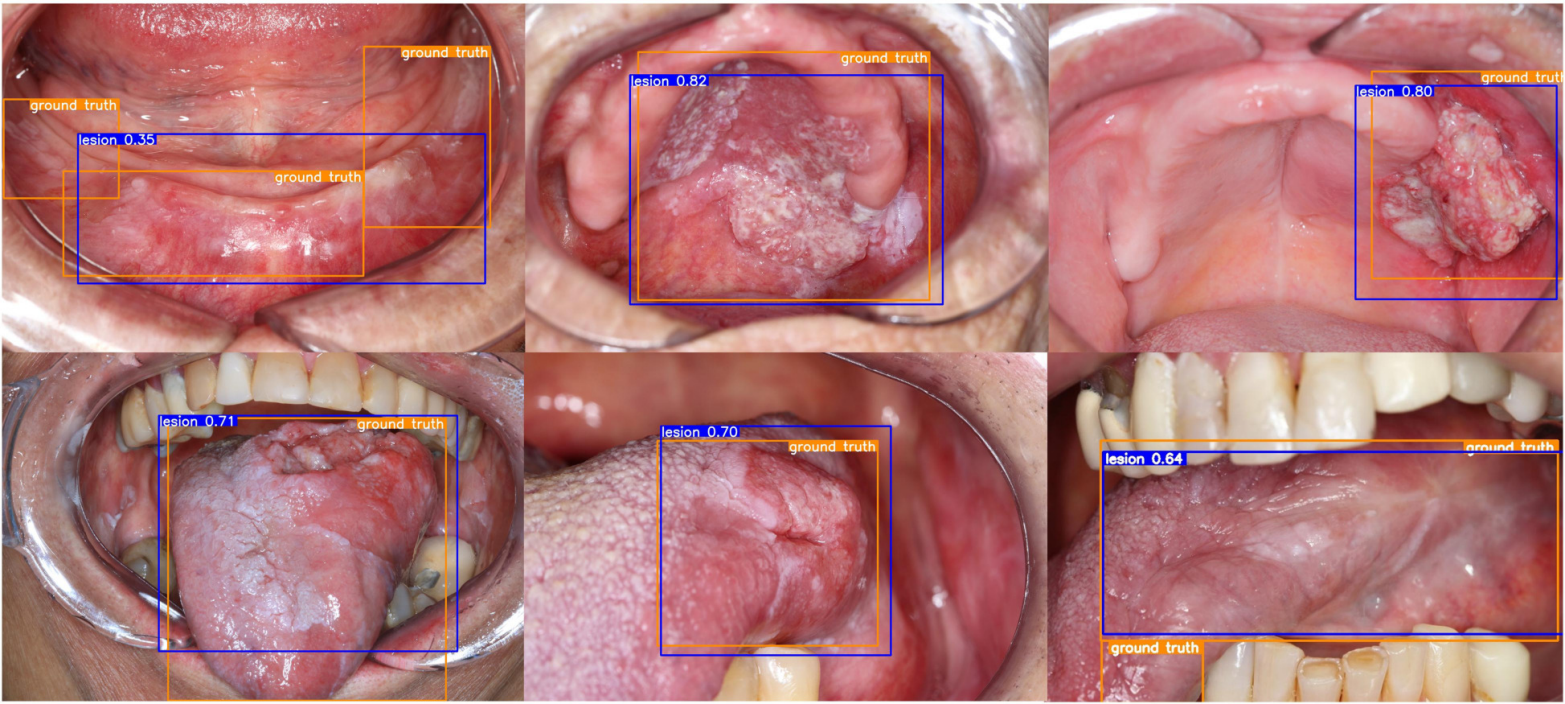
Lesion detection results with YOLO on the test set. Note the ground truth bounding boxes in orange and the generated bounding boxes in blue with the correspondent confidence scores.

The YOLOv11 models achieved variable performance on the lesion detection task, with mAP50 values ranging from 0.718–0.820 across different augmentation strategies. The best-performing configuration used Albumentations with slight blur, grayscale conversion, CLAHE (34), and minimal geometric transformations like an 80-degree rotation and a small perspective shift (0.001), achieving the highest mAP50 (0.820), precision (0.897), and F1-score (0.813). Although this setup yielded a slightly lower recall (0.744) compared to some other models, the substantial gains in precision and the resulting harmonic mean captured by the F1-score indicate a favorable trade-off (Table 2). Visual inspection of detection outputs confirmed consistent localization of lesions, as illustrated by bounding boxes and confidence scores over diverse oral cavity images. These results demonstrate that carefully tuned augmentations, especially rotation and limited perspective distortion, contributed to improved detection accuracy in the test set derived from the baseline dataset (Figure 1).

**Table 2:** YOLO11 results for lesion detection (one-class) using the baseline dataset.

Model	Augmentations					Test set metrics (baseline dataset)			
	Albumentations	Close_mosaic	Degrees	Perspective	Flipud	mAP50	Precision	Recall	F1-Score
YOLOv11n	True	0	0.0	0.0	0.0	0.767	0.811	0.718	0.761
YOLOv11n	True	0	80.0	0.0	0.5	0.784	0.841	0.748	0.792
YOLOv11n	True	0	0.0	0.0	0.5	0.743	0.763	**0** **.** **782**	0.772
YOLOv11n	True	50	0.0	0.0	0.5	0.776	0.831	0.758	0.792
YOLOv11n	True	100	0.0	0.0	0.5	0.765	0.775	0.718	0.745
YOLOv11s	True	0	80.0	0.0	0.5	0.766	0.724	0.740	0.732
YOLOv11m	True	0	80.0	0.0	0.5	0.752	0.720	0.692	0.705
YOLOv11l	True	0	80.0	0.0	0.5	0.718	0.713	0.679	0.695
YOLOv11n	False	0	80.0	0.0	0.5	0.788	0.766	0.754	0.760
YOLOv11n	True	0	80.0	0.001	0.5	**0** **.** **820**	**0** **.** **897**	0.744	**0** **.** **813**

Albumentations: image augmentation library used to apply transformations; Close_mosaic: augmentation strategy that merges images into a single composite; Degrees: angle range applied for random rotation augmentation; flipud: vertical flipping; mAP: mean average precision; Perspective: perspective transformation applied to images during augmentation.

**In bold:** best metrics.

### 3.2 Classification

The MobileNetV2 models trained for lesion classification showed notable improvements in performance when both horizontal and vertical flipping augmentations were applied. Specifically, the model trained with a learning rate of 0.0001 and both flipping augmentations achieved the highest accuracy of 0.846 (95% CI: 0.756–0.923), precision of 0.871 (95% CI: 0.817–0.933), recall of 0.846 (95% CI: 0.756–0.923), and F1-score of 0.844 (95% CI: 0.756–0.923), as well as the second-best AUC-ROC of 0.852 (95% CI: 0.759–0.941). Although it did not achieve the top AUC-ROC score, the difference was minimal (only 0.004), indicating that the overall discriminative ability of the model remained virtually unaffected. In comparison, omitting vertical flipping resulted in lower metrics across all evaluation measures, indicating that combining horizontal and vertical flipping contributed substantially to better generalization performance on the test set derived from the baseline dataset. Interestingly, reducing the learning rate to 0.00001 slightly decreased all performance metrics, suggesting that a moderately low learning rate coupled with diverse augmentations is optimal for this classification task (Table 3). After selecting the best MobileNetV2 configuration (Model 2, see Table 3) we further conducted an external validation which yielded an accuracy of 0.850 (0.798–0.902), precision of 0.866 (0.822–0.912), recall of 0.850 (0.798–0.902), F1-score of 0.851 (0.799–0.902), and an AUC-ROC of 0.935 (0.900–0.968).

**Table 3:** MobileNetV2 classification results on images cropped according to the experts' reference bounding box annotations using the baseline dataset.

Model	LR	Horizontal flipping	Vertical flipping	Accuracy (CI)	Precision (CI)	Recall (CI)	F1-score (CI)	AUC-ROC (CI)
1	0.0001	True	False	0.743 (0.654–0.846)	0.750 (0.656–0.852)	0.743 (0.654–0.846)	0.742 (0.647–0.846)	0.823 (0.725–0.913)
2	0.0001	True	True	**0.846** **(0.756–0.923)**	**0.871** **(0.817–0.933)**	**0.846** **(0.756–0.923)**	**0.844** **(0.756–0.923)**	0.852 (0.759–0.941)
3	0.00001	True	True	0.820 (0.731–0.897)	0.844 (0.772–0.909)	0.820 (0.731–0.897)	0.818 (0.727–0.897)	**0.856** **(0.755–0.928)**

Values are presented as mean (95% confidence interval) calculated via bootstrap with *N* = 1,000 resamples. LR, learning rate.

**In bold:** best metrics.

### 3.3 Two-step pipeline

For the two-step pipeline approach, we selected the best-performing MobileNetV2 model (Model 2, see Table 3), where the classifier was applied to image crops generated by the best-performing YOLOv11n (Table 2), applying a methodology similar to that reported by Fu et al. (35). We additionally computed the performance of the two-step pipeline in the external validation dataset (Table 4).

**Table 4:** Classification results of the best-performing MobileNetV2 model (Model 2) applied to image crops guided by the bounding box detections of the best-performing YOLOv11n.

Datasets	Accuracy (CI)	Precision (CI)	Recall (CI)	F1-score (CI)	AUC-ROC (CI)
Baseline dataset^a^	0.784 (0.696–0.863)	0.793 (0.712–0.872)	0.784 (0.696–0.863)	0.784 (0.696–0.863)	0.811 (0.712–0.889)
External dataset	**0.863** **(0.806–0.914)**	**0.879** **(0.831–0.928)**	**0.863** **(0.806–0.914)**	**0.866** **(0.807–0.916)**	**0.934** **(0.883–0.975)**

Values are presented as mean (95% confidence interval) calculated via bootstrap with *N* = 1,000 resamples.

**In bold:** best metrics.

a Test set derived from the baseline dataset (FOP-UNICAMP).

**Figure 2:**
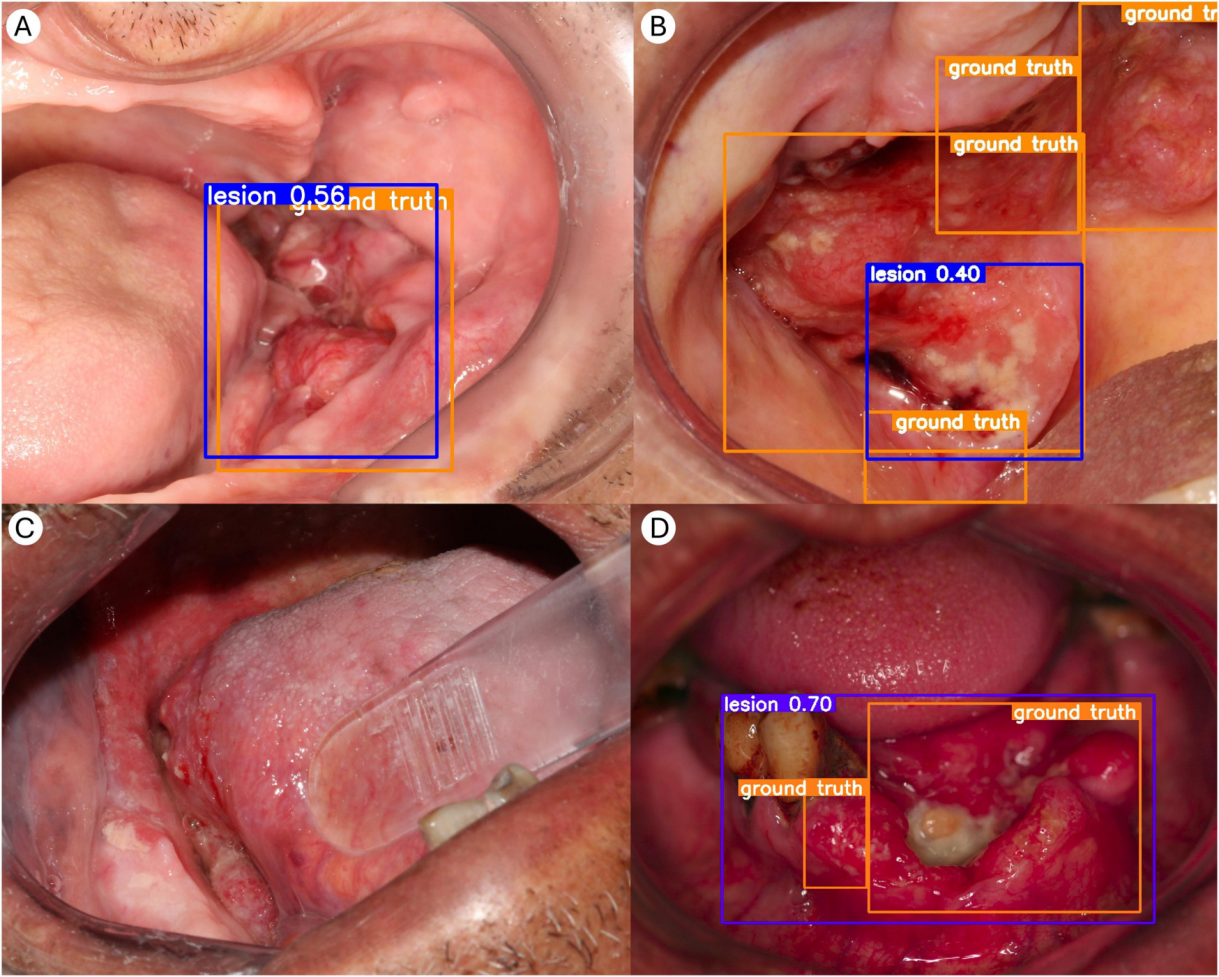
Visual inspection of YOLO's inference outputs. **(A)** Example of an optimal detection, with near-perfect overlap between the reference standard (ground truth, in orange) and the YOLO detection bounding box. **(B)** In some cases, the experts' reference bounding box annotations consisted of multiple boxes to encompass the entire lesion due to its size and arrangement. The single bounding box generated by the object detection model does not always cover the entire lesion. This suggests that YOLO may have difficulty predicting such lesions, as most images can typically be delineated with only one bounding box. Consequently, only the detected region (in blue) is forwarded to the MobileNetV2 classifier. This limited context may lead to misclassification in certain cases. **(C)** Example of an image in which the detector failed to identify the lesion. Of the 132 images in the external validation set, 23 (17.4%) did not yield a detection box. In such cases, these images were not forwarded to the classifier. **(D)** Example of a single large bounding box detected by YOLO, encompassing the lesional area with multiple annotated regions. Although the detection is visually and clinically accurate, the mAP score is penalized due to the presence of multiple annotated boxes, illustrating a potential discrepancy between visual performance and quantitative evaluation.

The visual representations are particularly important, as relying solely on YOLO's evaluation metrics may not fully reflect the model's performance. Examining the detection results themselves provides complementary insights that enhance model interpretability (Figure 2).

### 3.4 t-SNE plot

**Figure 3:**
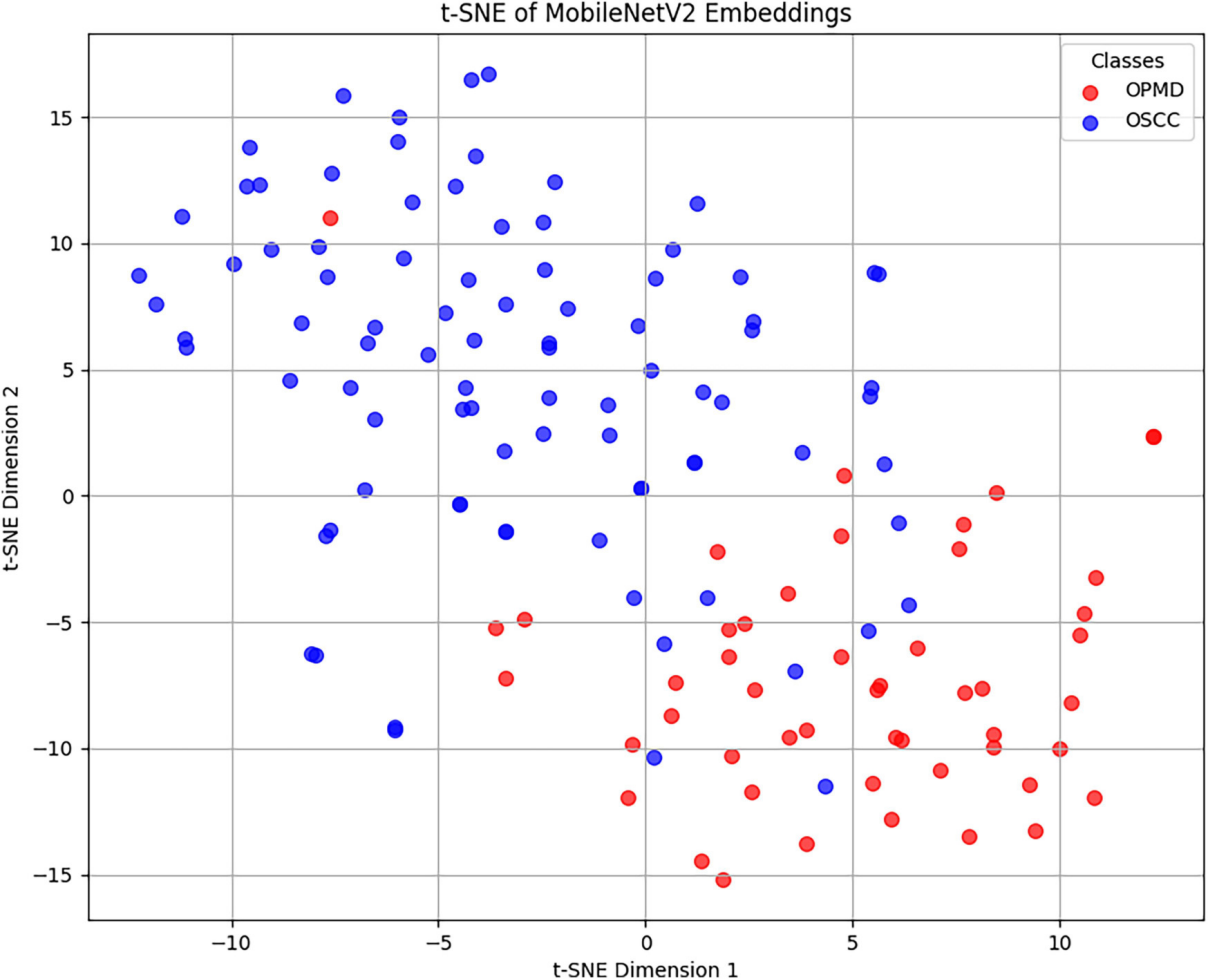
t-SNE visualization of feature embeddings in the external validation dataset. Each point represents an image projected into a 2-dimensional space, where similar embeddings (i.e., similar learned features) are mapped closer together. The points are color-coded by their true class (reference standard or “ground truth”): red for oral potentially malignant disorders (OPMD) and blue for oral squamous cell carcinoma (OSCC). The interpretation is that certain OSCC cases (blue points) exhibit such close clinical similarity to OPMDs that they overlap with the red points in the central and lower regions of the plot.

We further employed t-SNE to visualize the feature embeddings extracted by the two-step approach (YOLOv11n + MobileNetV2) applied to the external validation dataset, projecting them into a two-dimensional space to qualitatively assess the learned representations. The resulting plot (Figure 3) reveals a partial separation between OPMD and OSCC samples. This separation is evidenced by the formation of distinct, localized clusters predominantly populated by a single class. However, the model's ability to perfectly discriminate between classes is challenged by a significant area of overlap in the central and lower regions of the plot, where embeddings from both classes are intermingled. This visualization provides crucial insight into the model's performance: the partial clustering shows that MobileNetV2 has successfully captured discriminative, class-specific features. Conversely, the substantial overlap in the t-SNE plot, where points colored by their true diagnoses intermingle, reveals the intrinsic ambiguity in the dataset rather than a model shortcoming. This latent ambiguity reflects the real-world diagnostic challenge posed by borderline cases, where lesions from both classes share highly similar visual characteristics, leading to convergent feature representations. Consequently, classification errors often occur within this ambiguous region. The visualization thus explains the model's performance ceiling, indicating that some misclassifications stem from the inherent complexity and similarity of the lesions, rather than a failure of the model to learn.

## 4 Discussion

This study aimed to develop detection and classification algorithms to assist in oral cancer screening through a mobile application incorporating standardized clinical photography. Our results are consistent with prior research indicating that pre-trained weights, particularly from COCO, can accelerate model convergence and improve detection performance (12–15) (Supplementary Table S2). However, in line with findings by Welikala et al. (16), our observations also highlight that ImageNet pre-training may offer superior feature transfer for clinical images, likely due to its broader diversity and richer low-level representations. Despite leveraging established architectures such as YOLO and Faster R-CNN and applying common practices including data augmentation and consensus-based annotations, our approach remains constrained by the inherent subjectivity in lesion labeling, and the challenges of detecting small or subtle lesions, especially in OPMDs. Nonetheless, the comparable or superior performance of AI models relative to clinicians reported in the literature underscores the potential of such tools to augment early detection of OSCC, particularly when integrated into accessible mobile platforms aimed at supporting routine clinical workflows.

Our two-step pipeline follows a methodology similar to that reported by Fu et al. (35) which implemented backbone networks for detection and classification to localize lesions within clinical photographs by drawing bounding boxes. These cropped regions were subsequently used as input to the classifier. Unfortunately, our results are not directly comparable to those of Fu et al., as their work did not report detection metrics. The performance of our two-step pipeline on the external validation dataset [AUC = **0.934** (95% CI: **0.883–0.975**)] was comparable to the results reported by Fu et al., [AUC = **0.935** (95% CI: **0.910–0.957**)] suggesting that our approach achieves a similar level of discriminative ability despite differences in dataset composition and preprocessing protocols. The consistent results from external validation reinforce the potential generalizability of our pipeline across independent datasets. It is important to note that YOLO detections may present inherent limitations, such as bounding boxes that do not precisely encompass the lesion region or, in some instances, a complete omission of the lesion. Therefore, these factors must be considered when interpreting the performance of the two-step pipeline classifier, as they may affect the overall evaluation of the proposed approach.

As well as the majority of published studies (12–15) we also applied COCO weights to initialize models in their object detection tasks, enabling faster convergence and improved performance by leveraging pre-trained knowledge from a large and diverse dataset. Only one previously published study (16) pre-trained their model on ImageNet and the superior recall and F1-score achieved suggest that ImageNet may provide more effective feature transfer for clinical image analysis than COCO, likely due to its broader visual diversity and low-level representation capacity. Despite this, we cannot affirm that pre-trained weights guarantee better results, since models, datasets, and categories are different in each study. Other factors such as data augmentation, class balancing, and IoU threshold also influence the results.

It is well known that image labeling (lesion delineation or bounding box placement) may suffer from the subjectivity of the clinical exam, transferring this bias to the models if only one labeler is involved in data preparation. The largest area of diagnostic overlap (i.e., the most frequent area of intersection) is often defined as the ground truth for model training, (13–15) though combined annotations from multiple experts also represent a robust alternative approach (16). In our protocol, a consensus between two labelers during annotation generated a single bounding box to train the models (with the exception of some lesions due to their size and arrangement), reducing one step of complexity in the engineering process.

After an extensive literature review, we identified five studies (12–16) that implemented object detection models using various architectures. These included different versions of YOLO (12–14), Faster R-CNN (13–16), RetinaNet (13), and CenterNet2 (14). Among these, YOLO was the most frequently used architecture, being adopted in all studies except (15, 16). The popularity of YOLO can be attributed to its direct regression approach to bounding boxes, which aligns with end-to-end object detection goals, its compact model size, and low implementation complexity, making it particularly suitable for real-time applications and mobile deployment (26). However, a notable limitation is that some versions of YOLO tend to lose accuracy when detecting small objects (36).

With respect to sampling strategies, the baseline dataset was partitioned into an 80:10:10 ratio, with an independent test set derived from the baseline dataset to assess generalization. Furthermore, to reinforce the robustness of our approach, an external validation was performed using a separate dataset, in line with the recommendations of Cerdá-Alberich et al. and Tejani et al. (24, 25). This 80:10:10 split is consistent with the approach of Tanriver et al. (12), although it is not the most commonly used sampling strategy in published object detection studies. Five-fold cross-validation remains a good alternative when data are limited (13–15), but strategies such as bootstrapping, ensemble learning, label smoothing, stratified/nested cross-validation, and early stopping should be applied to account for data heterogeneity, label noise, and potential overfitting to ensure the robustness and generalizability of the models (37).

For object detection evaluation, the recommended metrics to assess a model's ability to correctly identify meaningful regions include precision, recall (sensitivity), F1-score, Intersection over Union (IoU), and mean Average Precision (mAP). Among these, mAP is the most comprehensive, as it evaluates how well the model detects objects across all classes, at multiple confidence thresholds, and is well-suited for comparing different object detection models as well as assessing performance across object sizes and complexities (28). Despite its relevance, only one previously published study (12) and the present research have reported this metric. It is also important to note that IoU is a particularly strict metric, as it measures how well the predicted bounding box overlaps with the reference (ground truth), effectively defining what counts as a correct detection. This often leads to penalization of partially accurate predictions, especially in cases of small or irregularly shaped lesions.

Warin et al. (14) compared the sensitivity of AI models with that of oral and maxillofacial surgeons in the detection of OSCC. Surprisingly, their least performant model, CenterNet2, still outperformed the surgeons in detecting OSCC. On the other hand, surgeons maintained higher sensitivity in detecting OPMD compared to the models. This may be explained by the fact that OPMDs encompass a heterogeneous group of lesions with often subtle features, which can be easily confused with other conditions and may be difficult to recognize, even for experienced clinicians. There is also evidence suggesting that fast detection models may not be ideal for capturing the nuanced features of OPMDs.

A strength of the present work is that the baseline dataset spans 25 years (2000–2025), adding potential variability in image acquisition (e.g., devices, lighting), which may improve model robustness (if consistently annotated). The classes are roughly balanced, avoiding the need for heavy resampling or weighting (38). Still, we performed data augmentation on the training set to improve model robustness, following methods established in previous studies. This approach was necessary due to the limited size of our dataset. Concerning data variability, we included incipient and conventional OSCC and different OPMD subtypes, which is valuable for clinical realism. The small number of images in certain subclasses mirrors the real-world scenario, where some lesion types are inherently less frequent.

Our study shares common limitations found in most deep learning (DL) work in the health domain: DL models typically perform better with thousands of labeled examples. Although the dataset is limited in size, several measures were implemented during training to mitigate this constraint: data augmentation, and a rigorous training regimen utilizing a dedicated training split and a validation split to monitor loss throughout the epochs, enabling early stopping if necessary. The model's performance was then evaluated on a held-out test set. Furthermore, the model was validated on an external dataset acquired under different conditions, including capture parameters, sensors, and geolocation. The high performance observed on this external set strongly suggests that the model generalized effectively and did not overfit to the training data. It is important to note that a proportion of images (17.4%) did not generate a detection box and were therefore excluded from the classification stage. This limitation should be taken into account when interpreting the results, as it may introduce bias by reducing the effective sample size and potentially underrepresenting certain lesion presentations. Another limitation of this study is that the image augmentation parameters were based on default values from the YOLO framework rather than being optimized for our specific dataset; we plan to address this in future work by conducting a detailed ablation study to evaluate and refine these hyperparameters. Although explainability methods were not applied in this study, the YOLO-based detection stage inherently provides an intuitive understanding of the model's behavior through object localization. Given that the MobileNetV2 classifier was applied exclusively to cropped regions of interest, *post-hoc* attribution techniques like Grad-CAM would offer limited incremental insight in this context. Nonetheless, we fully acknowledge the critical role of model interpretability for clinical adoption and intend to investigate complementary explainability approaches, such as Gradient-weighted Class Activation Mapping (Grad-CAM) (41, 42) and SHapley Additive exPlanations (SHAP) (43), in future work.

To date, no technology has provided definitive evidence of improved sensitivity or specificity for oral cancer screening when compared to conventional oral examination (39). By definition, screening refers to the systematic application of a test or procedure to asymptomatic individuals in order to detect disease at an early, treatable stage (3). Therefore, AI systems that aim to classify lesions already identified during the intraoral clinical exam cannot be considered true screening tools. Instead, they function as diagnostic support systems, assisting clinicians in the evaluation of lesions that have already been detected. Additionally, there is not enough evidence that these support systems alter the course of the disease or reduce mortality. Even in the context of early detection, whether using AI-based systems or not, leukoplakia often recurs over time despite the different treatment modalities employed (e.g., surgery or CO₂ laser) (40). It is also unclear whether any meaningful changes in the disease course would occur even if we could accurately identify which patients will eventually develop malignant transformation.

## 5 Conclusion

This study serves as an exploratory or proof-of-concept two-step pipeline for the early detection of oral cancer and the classification of OPMD and OSCC. Future work will focus on a prospective study in which patients are diagnosed with the assistance of a mobile application, offering a real-time, low-cost, non-invasive, and user-friendly support system for oral diseases diagnosis.

## Data Availability

The datasets used and/or analyzed during the current study are available from the corresponding author on reasonable request. All authors agree to be accountable for any aspects of the work and we ensure that questions related to the accuracy or integrity of any part of the work are appropriately investigated and resolved.
