# Supplementary material for: Two-step pipeline for oral diseases detection and classification: a deep learning approach
Source: Front Oral Health. 2025 Oct 27;6:1659323. doi: 10.3389/froh.2025.1659323 (PMC12599429; doi:10.3389/froh.2025.1659323)
Supplement: Supplementary file 1 [file Table1.docx]

Supplementary Material

# Supplementary Tables

**Supplementary Table 1.** Methodological Quality Assessment Using AI-Specific Checklists

| **Checklist for Artificial Intelligence in Medical Imaging (CLAIM)** (Tejani et al., 2024) | | | |  |
| --- | --- | --- | --- | --- |
| TITLE/ABSTRACT |  | 1 | Identification as a study of AI methodology, specifying the category of technology  used (eg, deep learning) | Yes |
| ABSTRACT |  | 2 | Summary of study design, methods, results, and conclusions | Yes |
| INTRODUCTION |  | 3 | Scientific and/or clinical background, including the intended use and role of the AI  approach | Yes |
|  |  | 4 | Study aims, objectives, and hypotheses | Yes |
| METHODS | *Study Design* | 5 | Prospective or retrospective study | Yes |
|  |  | 6 | Study goal | Yes |
|  | *Data* | 7 | Data sources | Yes |
|  |  | 8 | Inclusion and exclusion criteria | Yes |
|  |  | 9 | Data preprocessing | Yes |
|  |  | 10 | Selection of data subsets | Yes |
|  |  | 11 | De-identification methods | Yes |
|  |  | 12 | How missing data were handled | NA |
|  |  | 13 | Image acquisition protocol | Yes |
|  | *Reference Standard* | 14 | Definition of method(s) used to obtain reference standard | Yes |
|  |  | 15 | Rationale for choosing the reference standard | Yes |
|  |  | 16 | Source of reference standard annotations | Yes |
|  |  | 17 | Annotation of test set | Yes |
|  |  | 18 | Measures of inter- and intrarater variability of features described by the annotators | Yes |
|  | *Data Partitions* | 19 | How data were assigned to partitions | Yes |
|  |  | 20 | Level at which partitions are disjoint | Yes |
|  | *Testing Data* | 21 | Intended sample size | Yes |
|  | Model | 22 | Detailed description of model | Yes |
|  |  | 23 | Software libraries, frameworks, and packages | Yes |
|  |  | 24 | Initialization of model parameters | Yes |
|  | *Training* | 25 | Details of training approach | Yes |
|  |  | 26 | Method of selecting the final model | Yes |
|  |  | 27 | Ensembling techniques | NA |
|  | Evaluation | 28 | Metrics of model performance | Yes |
|  |  | 29 | Statistical measures of significance and uncertainty | Yes |
|  |  | 30 | Robustness or sensitivity analysis | Yes |
|  |  | 31 | Methods for explainability or interpretability | NA |
|  |  | 32 | Evaluation on internal data | Yes |
|  |  | 33 | Testing on external data | Sim |
|  |  | 34 | Clinical trial registration | NA |
|  | Data | 35 | Numbers of patients or examinations included and excluded | Yes |
|  |  | 36 | Demographic and clinical characteristics of cases in each partition | NA |
|  | *Model Performance* | 37 | Performance metrics and measures of statistical uncertainty | Yes |
|  |  | 38 | Estimates of diagnostic performance and their precision | Yes |
|  |  | 39 | Failure analysis of incorrectly classified cases | Yes |
| DISCUSSION |  | 40 | Study limitations | Yes |
|  |  | 41 | Implications for practice, including intended use and/or clinical role | Yes |
| OTHER INFORMATION |  | 42 | Provide a reference to the full study protocol or to additional technical details | Yes |
|  |  | 43 | Statement about the availability of software, trained model, and/or data | Yes |
|  |  | 44 | Sources of funding and other support; role of funders | Yes |
| **Must AI Criteria-10 checklist (MAIC–10)** (Cerdá-Alberich et al., 2023) | | | | |
| 1. Clinical need | Introduction | | The study is clearly put into context by describing the target clinical problem and  any previous approaches in the literature | Yes |
| 2. Study design | Materials and methods | | The type of study (observational/interventional, single/multicentre) and inclusion/  exclusion criteria are explicitly described, and a sample size estimate is given | Yes |
| 3. Safety and privacy | Materials and methods | | ELSI (ethical, legal, social implications), specifically including ethics committee  approval and data de-identification issues, are discussed | Yes |
| 4. Data curation | Materials and methods | | Data extraction, cleaning, and transformation methods, including image pre-processing  steps, are clearly described | Yes |
| 5. Data annotation | Materials and methods | | The ground truth reference is defined and the annotation process, including measures  of inter/intra-observer variability, is described | Yes |
| 6. Data partitioning | Materials and methods | | Methods and criteria for data set splitting into train-tune-test-validation sets are  indicated | Yes |
| 7. AI model | Materials and methods, results | | The AI model building methodology is sufficiently detailed by including used technologies  (software and hardware), training–tuning–testing methods, performance  metrics, and resulting AI model architecture | Yes |
| 8. Robustness | Results, discussion | | The generalizability of the AI model in real-world conditions is explicitly discussed | Yes |
| 9. Explainability | Discussion | | The interpretability of the model (including the use of uncertainty or confidence  metrics) is explicitly discussed | Yes |
| 10.Transparency | Discussion | | Any possibility of access to original data sets and source code is clearly stated.  Financing and conflicts of interest are detailed | Yes |

**Supplementary Table 2**. Summary of related studies using object detection frameworks

| **Author, year (citation)** | **Images (n)** | **Categories (n)** | **Models** | **mAP50** | **Precision** | **Recall (sensitivity)** | **F1-score or Dice coefficient** | **AUROC** |
| --- | --- | --- | --- | --- | --- | --- | --- | --- |
| Tanriver et al. 2021 (Tanriver et al., 2021) | 652 | Benign (274), OPMD (248), or carcinoma (162) | YOLOv5s | 0.920 | NI | NI | NI | NI |
|  |  |  | YOLOv5m | 0.896 | NI | NI | NI | NI |
|  |  |  | YOLOv5l | 0.951 | NI | NI | NI | NI |
|  |  |  | YOLOv5x | 0.902 | NI | NI | NI | NI |
|  |  |  | YOLOv5x + TTA | 0.940 | NI | NI | NI | NI |
|  |  |  | YOLOv5s & 5m  ensemble | 0.923 | NI | NI | NI | NI |
| Warin et al. 2021 (Warin et al., 2021) | 700 | Normal (350) and OSCC (350) | Faster R-CNN | NI | 0.7667 | 0.8214 | 0.7931 | 0.79 |
| Warin et al. 2022a (Warin et al., 2022) | 980 | Non-pathological (300), OPMD (315) and OSCC (365) | Faster R-CNN | NI | OPMD: 0.60  OSCC: 0.84 | OPMD: 0.71  OSCC: 0.90 | OPMD: 0.65  OSCC: 0.87 | OPMD: 0.64  OSCC: 0.88 |
|  |  |  | YOLOv5 | NI | OPMD: 0.74  OSCC: 0.88 | OPMD: 0.39  OSCC: 0.86 | OPMD: 0.51  OSCC: 0.87 | OPMD: 0.34  OSCC: 0.84 |
|  |  |  | RetinaNet | NI | OPMD: 0.92  OSCC: 0.98 | OPMD: 0.57  OSCC: 0.82 | OPMD: 0.70  OSCC: 0.89 | OPMD: 0.55  OSCC: 0.81 |
|  |  |  | CenterNet2 | NI | OPMD: 0.49  OSCC: 0.64 | OPMD: 0.60  OSCC: 0.92 | OPMD: 0.54  OSCC: 0.76 | OPMD: 0.58  OSCC: 0.91 |
| Warin et al. 2022b (Warin et al., 2022) | 600 | Normal (300), OPMD (300) | Faster R-CNN | NI | 0.7969 | 0.81 | 0.8031 | 0.7434 |
|  |  |  | YOLOv4 | NI | 0.5238 | 0.5238 | 0.5238 | 0.4418 |
| Welikala et al. 2021 (Welikala et al., 2020) | 2,155 | Lesion | Faster R-CNN | NI | 0.4661 | 0.3716 | 0.4135 | NI |
|  |  | Referral, Non-referal | Faster R-CNN |  | 0.6715 | 0.9388 | 0.7830 | NI |
| **Present work** | **773** | **Lesion** | **YOLO11n** | **0.813** | **0.860** | **0.789** | **0.813** | **NR*** |

AUROC: area under the receiver operator characteristics; OPMD: oral potentially malignant disorder; OSCC: oral squamous cell carcinoma;

YOLO: you only look once; TTA: test-time augmentation; NI: not informed; mAP50: mean average precision calculated at a fixed IoU threshold of 0.5; *Not reported (one-class problem)
